# How Health Care Workers Can Manage Digital Fatigue

**DOI:** 10.2196/104196

**Published:** 2026-06-15

**Authors:** Sara Novak

**Keywords:** burnout, professional, electronic health records, health personnel, medical informatics, occupational stress, user-computer interface, workload, digital fatigue

## Abstract

One of the key aims of the digitization of health care is reducing burden on health care workers and health care systems. In this *News and Perspectives* article, JMIR Correspondent Sara Novak reports on one of its paradoxical consequences: digital fatigue.

**Key Takeaways:**

- While health care’s digital transformation has the potential to improve care, it can also make more work for health care workers, contributing to exhaustion and burnout.
- Alerts and redundant messages need to be streamlined, so that health care workers aren’t overloaded.
- The digital workload needs to be a recognized aspect of the workday.

Health care’s digital transformation has the potential to improve care while also easing the workload on physicians, nurses, and health care workers by making services accessible, streamlining processes, and automating time-consuming tasks. But digitization can also increase workload by forcing health care workers to learn new tools and systems and shoulder the burden of integrating and implementing them.

As health care workers become increasingly dependent on digitization through electronic health records (EHRs), data dashboards, and health information exchanges, it’s important for health care institutions and for the workers themselves to establish personal strategies to manage digital fatigue and the overwhelm, exhaustion, and burnout that digitization can cause.

“Technology promised to ‘give you time back,’ only for physicians to discover that it often reallocated that time from patients to screens,” said Hassan Bencheqroun, MD, MBA—a pulmonologist, critical care physician, and assistant professor of medicine at the University of California, Riverside.

## The Causes of Digital Health Care Fatigue

Health care workers are already expected to manage patient care, time pressure, and the emotional strain of taking care of sick patients. Digital fatigue can add another layer to this already potent emotional strain.

“Digital fatigue in this context is not only about screen time or clicks; it’s about misaligned promises and unbalanced trade-offs. The result is a growing sense that we are supervising machines rather than practicing medicine,” said Bencheqroun.

When health care workers are constantly having to interact with and respond to messages and alerts in the EHR, it takes away from the amount of time that they can spend face-to-face with patients.

One study published in the journal *BMC Nursing* points to the emerging phenomenon of digital compassion fatigue (DCF)—a form of emotional exhaustion that results when health care workers provide empathic care through virtual platforms. DCF is defined by persistent emotional exhaustion after digital engagement, as well as cognitive overload, feelings of professional inadequacy, and a fear of a reduction in the quality of care. Health care workers feel like the time spent on digital platforms makes it impossible for them to spend adequate time with their patients.

Health care workers feel less connected to patients when they’re spending too much time on digital devices and also having less time to recover at the end of the day because they have to spend time outside of work hours responding to EHR alerts and messages, said Rachel Hoopsick, PhD—an assistant professor of health and kinesiology at University of Illinois, Urbana-Champaign, who studies digital fatigue in health care workers.

Hoopsick said that we’re dealing with a fee-for-service health care system, meaning that health care providers are paid for the services that they provide, including tests and office visits, so time spent with each patient is already limited. Adding to the administrative burden by having to learn new systems only adds fuel to the fire.

“It’s a cycle that contributes to the eventual burnout of healthcare workers,” said Hoopsick.

## Managing Digital Fatigue Like You Would Other Occupational Health Risks

Digital fatigue needs to be taken seriously, like you would any other occupational risk. This starts by reducing the low-hanging fruit, including low-value alerts that pop up within the EHR, said Hoopsick.

For example, one study published in the journal *Applied Clinical Informatics* noted that most of the allergy alert prompts given for medications were for non–life-threatening allergies. In addition, one-third of alerts were redundant. Research also suggests that clinicians often ignore repeated alert guidance, “suggesting the guidance is not clinically helpful nor likely to alter clinical management,” wrote the study authors.

**Figure:**
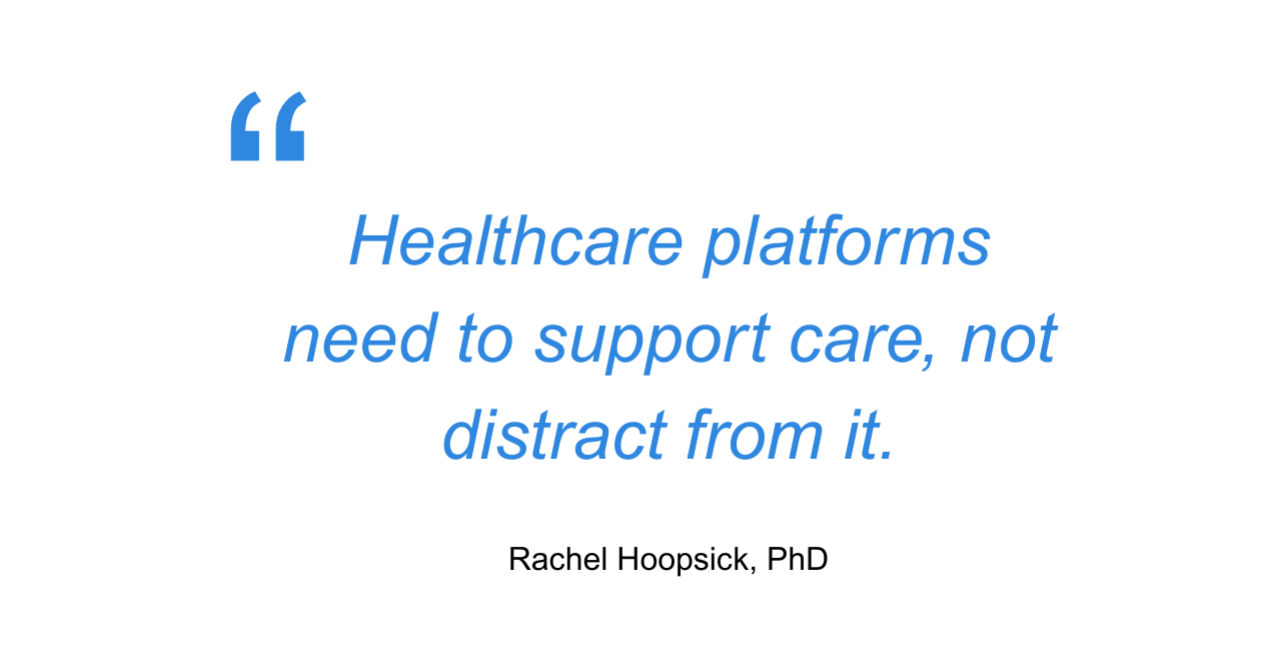


According to American Medical Association (AMA) guidance, other tools for reducing portal messages include annual automatic renewal of certain prescriptions and getting previsit labs, so results can be discussed at annual visits, rather than going back and forth on the EHR afterward.

The digital workflow also needs to be more team-based. For example, having a few members of the team designated to deal with inbox management, so that portal messages, medication refills, and administrative requests don’t pile up and result in after-hours workload. This way, the messages aren’t ignored and don’t end up unanswered.

Alternatively, the task of responding to messages can be separated individually. For example, the member of the team who orders a test should be responsible for communicating with the patient about it. If a team member is out of town, then another member involved with triage should accept responsibility while they’re away and vice versa, said Hoopsick.

Additionally, health care institutions need to recognize the digital workload as part of a health care worker’s tasks and not simply expect that it will be done outside of regular business hours, said Audrey Hai, PhD, MSW—an assistant professor at Tulane University in New Orleans who studies digital fatigue. She adds that in the digital age, much of the workload isn’t recognized by the institution, which has an impact on the caseload of each clinician.

“Institutions need to consider the 50 emails that you likely have to respond to when assigning patients. It’s part of the ‘invisible workload’ that’s become associated with digital care,” said Hai.

Additionally, when health care workers switch health care institutions or move to a new digital platform, health care institutions need to consider the training involved in learning a new system and not have an expectation that workers should teach themselves the platform, which is inefficient and takes time out of their workday. Not feeling competent in digital skills can also contribute to DCF, said Hai.

While it’s the health care institutions that need to bear the brunt of reducing digital fatigue, at the individual level, there are also a number of steps that health care workers can take. Hai recommends scheduling a digital detox break in your calendar every few days to allow for a few minutes away from all devices and screens.

Workers can also schedule email delivery, so that when coworkers or patients send you messages, you can schedule them to be delivered the next morning. That way, you don’t have to constantly check your phone past working hours, and you can be rested and ready to take on the next workday.

“Healthcare platforms need to support care, not distract from it,” said Hoopsick.

